# Flux-sum analysis identifies metabolite targets for strain improvement

**DOI:** 10.1186/s12918-015-0198-3

**Published:** 2015-10-29

**Authors:** Meiyappan Lakshmanan, Tae Yong Kim, Bevan K. S. Chung, Sang Yup Lee, Dong-Yup Lee

**Affiliations:** Bioprocessing Technology Institute, Agency for Science, Technology and Research (A*STAR), 20 Biopolis Way, #06-01 Centros, Singapore, 138668 Singapore; Metabolic and Biomolecular Engineering National Research Laboratory, Department of Chemical and Biomolecular Engineering (BK21 program), Korea Advanced Institute of Science and Technology (KAIST), Daejeon, 305-701 Republic of Korea; Center for Systems and Synthetic Biotechnology, Bioinformatics Research Center, Institute for the BioCentury, and Department of Bio and Brain Engineering, KAIST, Daejeon, 305-701 Republic of Korea; Department of Chemical and Biomolecular Engineering, National University of Singapore, 4 Engineering Drive 4, Singapore, 117576 Singapore; Synthetic Biology for Clinical and Technological Innovation (SynCTI), Life Sciences Institute, National University of Singapore, 28 Medical Drive, Singapore, 117456 Singapore

**Keywords:** Flux-sum, Constraints-based flux Analysis, Genome-scale metabolic modeling, Metabolic engineering, Strain design

## Abstract

**Background:**

Rational design of microbial strains for enhanced cellular physiology through *in silico* analysis has been reported in many metabolic engineering studies. Such *in silico* techniques typically involve the analysis of a metabolic model describing the metabolic and physiological states under various perturbed conditions, thereby identifying genetic targets to be manipulated for strain improvement. More often than not, the activation/inhibition of multiple reactions is necessary to produce a predicted change for improvement of cellular properties or states. However, as it is more computationally cumbersome to simulate all possible combinations of reaction perturbations, it is desirable to consider alternative techniques for identifying such metabolic engineering targets.

**Results:**

In this study, we present the modified version of previously developed metabolite-centric approach, also known as flux-sum analysis (FSA), for identifying metabolic engineering targets. Utility of FSA was demonstrated by applying it to *Escherichia coli*, as case studies, for enhancing ethanol and succinate production, and reducing acetate formation. Interestingly, most of the identified metabolites correspond to gene targets that have been experimentally validated in previous works on *E. coli* strain improvement. A notable example is that pyruvate, the metabolite target for enhancing succinate production, was found to be associated with multiple reaction targets that were only identifiable through more computationally expensive means. In addition, detailed analysis of the flux-sum perturbed conditions also provided valuable insights into how previous metabolic engineering strategies have been successful in enhancing cellular physiology.

**Conclusions:**

The application of FSA under the flux balance framework can identify novel metabolic engineering targets from the metabolite-centric perspective. Therefore, the current approach opens up a new research avenue for rational design and engineering of industrial microbes in the field of systems metabolic engineering.

**Electronic supplementary material:**

The online version of this article (doi:10.1186/s12918-015-0198-3) contains supplementary material, which is available to authorized users.

## Background

Previous microbial engineering for strain improvement was largely based on biological intuition and/or trial-and-error methods, such as random mutagenesis. However, with the recent advent of high-throughput experimental technologies and improved *in silico* modeling capabilities, there is growing interest in the application of the systems biology approach to metabolic engineering studies [1–3]. Notably, genome-scale metabolic models (GEMs) provide a convenient and cost-effective platform for systems biologists to carry out metabolic perturbation studies *in silico* and rationalize findings from high-throughput experiments. This practical utility consequently fueled the reconstruction of over 100 GEMs representing the metabolic organization of various organisms across all three domains of life [4, 5].

Specifically, *in silico* analysis of GEMs enables us to achieve the following goals: (1) interpreting high-throughput omics data, (2) aiding design of metabolic engineering strategies, (3) generating new testable hypotheses to gain knowledge of the biological system, (4) investigating inter-cellular and inter-species interactions and (5) understanding of complex genotype-phenotype relationships leading to discovery of emergent properties [6–9]. Among these, the successful application of GEM analysis to aid cellular metabolic engineering has been consistently reported in numerous studies ranging from simple *in silico* simulation of gene deletions [10, 11], to more sophisticated computational techniques such as OptKnock [12], OptReg [13], OptStrain [14], OptGene [15], flux response analysis [16], RobustKnock [17], flux scanning based on enforced objective flux [18], OptForce [19] and most recently, cofactor modification analysis [20] for identifying valid gene knockout, up-, down-regulation and cofactor engineering targets (see [21] for thorough review). These *in silico* methods share the common theme of identifying suitable genetically and environmentally perturbed conditions by optimizing the cellular objective, typically cell growth, under mass balance, reaction reversibility and flux capacity constraints. Basically, such a constraints-based technique mimics the wet-lab genetic engineering experiments by imposing relevant constraints on the metabolic reaction fluxes and can thus be considered “reaction-centric”. However, more often than not, these techniques have encountered the computational issue of combinatorial explosion due to consideration of multiple reaction perturbations. Hence, to deal with this limitation, an alternative method is needed to further expand our *in silico* capability of multiple reaction targets identification.

Recognizing that many metabolites are involved in multiple reactions, a “metabolite-centric” approach can potentially find individual metabolite targets implicating some combination of reaction flux constraints. In this way, the metabolite-centric flux-sum analysis (FSA) was initially utilized to evaluate the structural and evolutionary properties of cellular metabolism in *E. coli* [22, 23]. Later, the same concept was successfully utilized to compare and contrast the metabolic capabilities of *Z. mobilis* and *E. coli* [24]. Nonetheless, its potential applicability of identifying metabolic engineering targets for strain improvement remains unexamined. Therefore, in this study, we demonstrate the efficacy of FSA by applying it to *Escherichia coli* for increasing succinate and ethanol production, and reducing acetate formation as case studies.

## Results

### Flux-sum analysis for identifying metabolic engineering targets

In this work, we harness the previously presented computational technique, flux-sum analysis [22], to identify metabolite targets that will “force” the overproduction of desirable by-products or the reduction in formation of undesirable metabolites upon the attenuation or intensification of metabolite turnover, also known as “flux-sum”. The concept of “forcing” the desired metabolic behavior is similar to a previous study [19]. Briefly, first, the conventional constraints-based flux analysis problem is solved with biomass maximization as objective and the wild-type flux-sum of all metabolites are calculated as a reference. Second, the minimal and maximal flux-sum values of each metabolite are computed to determine the allowable range for attenuation and intensification from the reference state, respectively. Next, a MILP problem is sequentially solved for all metabolites to attain the maximum cell growth at various flux-sum perturbations between the calculated minimal and maximal values. Finally, using this growth values as additional minimum biomass production constraint, the same optimization problem is again solved for all metabolites to investigate whether the perturbation of a particular metabolite’s flux-sum improves the desired target compound production or not (see Methods). To demonstrate the applicability of this proposed framework, we apply it to the *E. coli* genome-scale metabolic model, thereby identifying the possible metabolite attenuation/intensification targets which can enhance the production of ethanol/succinate, and reduce the formation of toxic acetate. The list of identified best metabolite targets are summarized in Table 1 and will continue to examine each case in detail to gain a better understanding of their effects on cellular metabolism (see Additional file 1 for the target compound production profile under flux-sum perturbation of metabolites presented in Table 1).

**Table 1:** List of metabolic engineering targets

Objective	Target metabolites	Flux-sum perturbation	Experimental validation
Enhance ethanol production	Acetate	Attenuation	[25, 26]
	Acetylphosphate	Attenuation	[25, 26]
	Formate	Attenuation	N.A.
	6-Phospho-D-gluconate	Intensification	N.A.
	Erythrose 4-phosphate	Intensification	N.A.
	5,10-Methylenetetrahydrofolate	Intensification	N.A.
	Sedoheptulose 7-phosphate	Intensification	N.A.
Enhance succinate production	Pyruvate	Attenuation	[11]
	Glyoxylate	Intensification	[30, 31]
	Menaquinone/menaquinol	Intensification	N.A.
Minimize acetate production	Acetate	Attenuation	[34, 35]
	Acetylphosphate	Attenuation	[34, 35]
	3-Phosphoglycerate	Intensification	N.A.
	4-Methyl-2-oxopentanoate	Intensification	N.A.
	Isocitrate	Intensification	[36]
	Lactate	Intensification	[34]
	Ribulose 5-phosphate	Intensification	N.A.
	Succinic semialdehyde	Intensification	N.A.

### Ethanol production under flux-sum attenuation

**Fig. 1:**
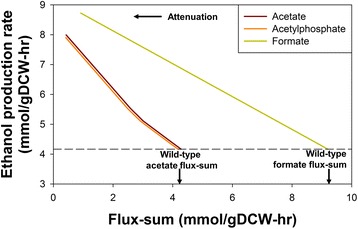
Ethanol production profile under metabolite flux-sum attenuation. The horizontal dashed line indicates the wild-type ethanol production value. Only the ethanol production profile corresponding to 3 metabolites are shown because flux-sum attenuation of the other metabolites do not yield such a desirable profile

**Fig. 2:**
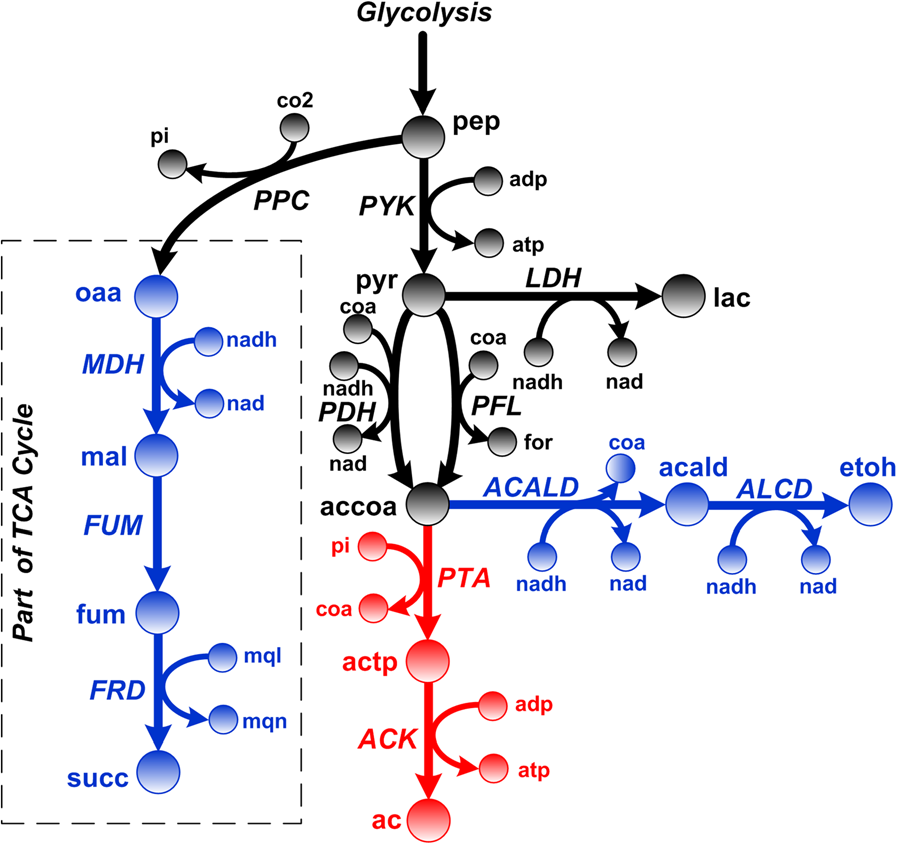
Mixed acid fermentation pathway of *E. coli*. The pathways illustrate that the formation of various organic acids and ethanol occurs in parallel, utilizing phosphoenolpyruvate as the common precursor. Blue colored reactions represent the biosynthesis of desired products, succinate and ethanol, whereas red color indicates the production of unwanted acetate. Abbreviation: ac, acetate; acald, acetaldehyde; accoa, acetyl-CoA; actp, acetylphosphate; etoh, ethanol; for, formate; lac, lactate; mal, malate; oaa, oxaloacetate; pep, phosphoenolpyruvate; pyr, pyruvate; ACALD, acetaldehyde dehydrogenase; ACK, acetate kinase; ALCD, alcohol dehydrogenase; FRD, fumarate reductase; FUM, fumarase; LDH, lactate dehydrogenase; MDH, malate dehydrogenase; PFL, pyruvate formate lyase; PPC, phosphoenolpyruvate carboxylase; PTA, phosphate acetyltransferase; PYK, pyruvate kinase

The flux-sums of individual metabolites are attenuated to investigate their effects on ethanol production. The ethanol production profile (Fig. 1) generated by FSA revealed that the attenuation of acetate and acetylphosphate flux-sums can “force” ethanol production rates to increase. It has also been demonstrated in previous experimental studies that deletion of the phosphate acetyltransferase gene (*pta*) resulted in improved ethanol production rate [25, 26]. The identification of acetate and acetylphosphate as flux-sum attenuation targets for ethanol overproduction is also an example of how parallel pathways compete for same carbon flux where the perturbation of one can positively favor the other (Fig. 2). This is similar to the previously identified essential metabolites located along parallel biosynthetic pathways which could cause sub-optimal distribution of metabolic fluxes and attenuated cell growth when their flux-sums were perturbed [22].

Another flux-sum attenuation target identified by FSA for the ethanol production is formate. Generally, acetyl-CoA in a cell can be synthesized in two ways. The pyruvate formate lyase (*PFL*) reaction yields acetyl-CoA and formate as the co-product. Another alternative is the pyruvate dehydrogenase (*PDH*) reaction that generates acetyl-CoA, CO_2_ and NADH. Therefore, attenuating formate flux-sum promotes acetyl-CoA formation through the *PDH* reaction, leading to increased NADH generation, which in turn drives the NADH-consuming alcohol dehydrogenase (*ALCD*) reaction towards ethanol production. Interestingly, it has been reported that a high flux through *PDH* can increase ethanol yield [27]. Hence, the construction of a *PFL*-repressed and *PDH*-overexpressed *E. coli* strain can be explored in future experimental validation to further improve ethanol production.

### Ethanol production under flux-sum intensification

Flux-sum intensification analysis identified several target metabolites such as 6-phospho-D-gluconate, erythrose-4-phosphate, ribulose-5-phosphate, sedoheptulose-7-phosphate and xylulose-5-phosphate that can improve ethanol production. Clearly, these metabolites are predominantly active in the NADPH-generating pentose phosphate pathway (PPP). Hence, flux-sum intensification of such metabolites will increase the formation of NADPH, which can be re-oxidized to NADP via the soluble NAD transhydrogenase (*UdhA*) reaction and simultaneously regenerate NADH. The elevated NADH regeneration rate can thus improve ethanol production. However, the *in vivo* activity of *UdhA* has been reported to be low [28] and this scenario of increased NADPH regeneration will most likely cause its accumulation that can possibly cause growth inhibition. Therefore, the simultaneous overexpression of *UdhA* and flux-sum intensification of PPP metabolites by overexpressing the glucose-6-phosphate dehydrogenase and gluconate dehydrogenase will be a promising strategy to increase ethanol production without the inhibitory effect.

Another target identified by the flux-sum intensification analysis is 5,10-methylenetetrahydrofolate (MLTHF), a key metabolite in folate metabolism. Examination of metabolic reactions involving MLTHF revealed that the metabolite can only be formed by glycine hydroxymethyltransferase reaction and the glycine cleavage reaction, where the latter reaction is coupled with NADH regeneration. Furthermore, FSA predicts that fluxes through both the reactions can be increased when MLTHF flux-sum is intensified. Thus, such a metabolic perturbation will have a positive effect on the ethanol-producing pathway which requires NADH as the co-substrate.

### Pyruvate decarboxylase insertion alters ethanol production profile

In previous metabolic engineering studies, it has been reported that the insertion of pyruvate decarboxylase (*PDC*) and alcohol dehydrogenase II genes can significantly improve ethanol production [29]. We can mimic this metabolic engineering strategy by inserting the *PDC* reaction into the GEM of *E. coli*, *i*AF1260 model and increasing *ALCD* flux, which is also equivalent to intensification of acetaldehyde flux-sum. Our simulation has shown that the intensification of acetaldehyde flux-sum in the wild-type *E. coli* GEM without the *PDC* reaction did not result in consistently increasing ethanol production. Thus, we carried out flux-sum intensification on the modified GEM of *E. coli* with the *PDC* reaction to further understand how *PDC* insertion can influence the ethanol production profile.

**Fig. 3:**
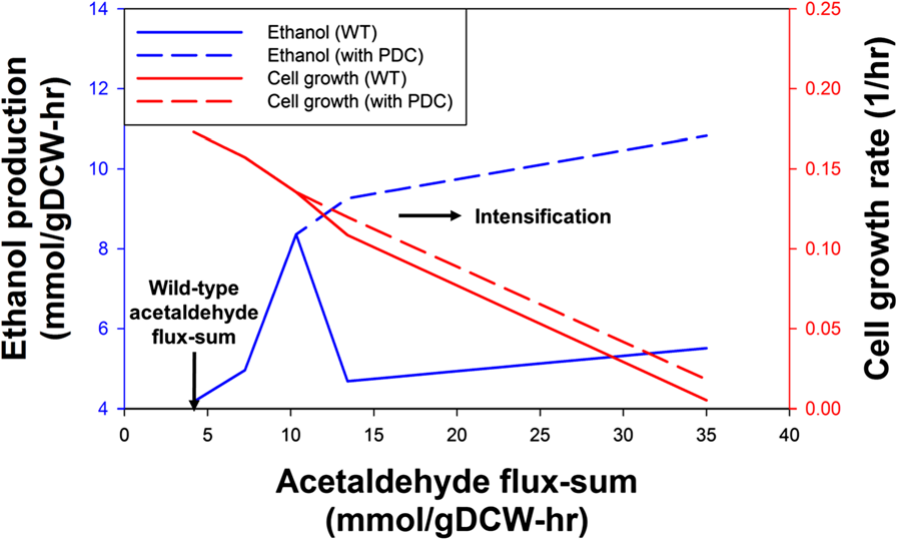
Ethanol production and cell growth profile under acetaldehyde flux-sum intensification. Wild-type acetaldehyde flux-sum was evaluated as 4.17 mmol/gDCW-hr using constraints-based flux analysis

**Fig. 4:**
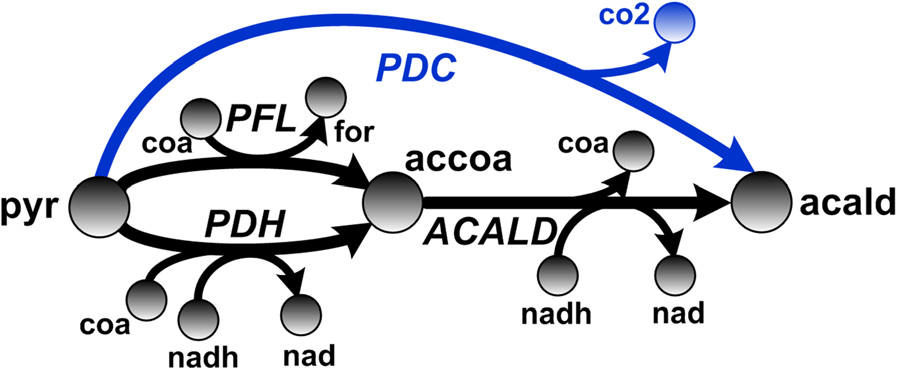
Metabolic reactions from pyruvate to acetaldehyde. Note that the black colored reactions are naturally present in wild-type *E. coli* whereas the blue colored pyruvate decarboxylase (*PDC*) is not

The new simulation results indeed show that addition of *PDC* reaction to the metabolic network improved the ethanol production profile under acetaldehyde flux-sum intensification (Fig. 3). In addition, a slightly higher cell growth rate can be achieved when the *PDC* reaction is available. This phenomenon of enhanced ethanol production due to such a metabolic engineering strategy can be understood by examining the reactions originating from pyruvate to acetaldehyde (Fig. 4). Wild-type conversion of pyruvate to acetaldehyde occurs via *PDH* or *PFL*, and then acetaldehyde dehydrogenase (*ACALD*). The addition of *PDC* allows the engineered *E. coli* strain to directly convert the pyruvate into acetaldehyde directly without forming the acetyl-CoA intermediate. Such direct conversion may positively assist the ethanol production as it circumvents the possible limitations which are associated with CoA regeneration in the *ACALD* step. Interestingly, intensification of acetaldehyde flux-sum beyond a certain point in wild-type decreases the ethanol production as the excess acetaldehyde gets converted into acetate via aldehyde dehydrogenase and then into acetyl-CoA via phosphotransacetylase (*PTA*) and acetate kinase (*ACK*), forming a cycle.

### Succinate production under flux-sum attenuation

**Fig. 5:**
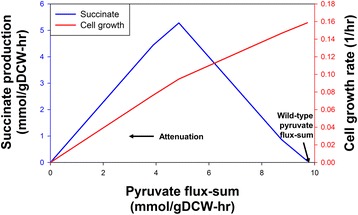
Succinate production and cell growth profiles under pyruvate flux-sum attenuation. Apart from pyruvate, the attenuation of other metabolite flux-sums does not cause an increase in succinate production rates. The wild-type succinate production rate is 0.06 mmol/gDCW-hr

Similar to the ethanol production case, we apply FSA to identify metabolite targets for strain improvement in the aspect of succinate production. The corresponding profile under metabolite flux-sum attenuation upon glucose uptake indicated that pyruvate is the only metabolite which can be targeted for enhancing succinate production (Fig. 5). This result has also been experimentally confirmed, demonstrating the improvement of succinate production through the reduction of total metabolic fluxes towards pyruvate by knocking out the genes of pyruvate-forming enzymes [11]. Moreover, our simulation also shows that succinate production can be negatively affected when pyruvate flux-sum is excessively attenuated (Fig. 5). Thus, we will further examine the corresponding flux distribution to gain a better understanding of how pyruvate flux-sum perturbation can alter the cellular metabolism.

Under the anaerobic condition, succinate dehydrogenase reaction is not utilized since oxygen is unavailable to regenerate the electron acceptor ubiquinone. Hence, instead of producing NAD through the ubiquinone-dependent NADH dehydrogenase reaction, NAD regeneration occurs mainly via ethanol-producing *ACALD* and *ALCD* reactions, which are part of the mixed acid fermentation pathway. Thus, by examining this pathway, we will attempt to explain how pyruvate flux-sum attenuation can lead to enhanced succinate production. From Fig. 2, it is clear that pyruvate is the precursor for acetate, ethanol, lactate and formate and synthesis. Pyruvate formation via the pyruvate kinase (*PYK*) and phosphoenolpyruvate/glucose phosphotransferase system (*PTS*) competes for the metabolic fluxes that could potentially flow towards succinate formation. Hence, attenuating pyruvate flux-sum favors the redirection of metabolic fluxes at the phosphoenolpyruvate branch towards succinate synthesis. Since pyruvate flux-sum attenuation also causes a concomitant decrease in NAD regeneration by *LDH*, *ACALD* and *ALCD*, the metabolic flux is preferably channeled towards succinate-producing fumarate reductase (*FRD*), where the NAD shortage can be compensated by the coupling of *FRD* with the menaquinone-dependent NADH dehydrogenase reaction. Therefore, the substitution of *LDH*, *ACALD* and *ALCD* by *FRD* as the major NAD regenerator can increase the succinate production under pyruvate flux-sum attenuation as depicted by the right section of the succinate production profile (Fig. 5). For the same reason, it may be possible that the *in vivo* deletion of *ptsG* and *pykFA* have given rise to more than eight-fold increase in succinate yield [11].

On the other hand, the left section of the profile (Fig. 5) can be explained by realizing that pyruvate flux-sum attenuation is also accompanied by decreased ATP regeneration due to lower *ACK* and pyruvate kinase (*PYK*) fluxes. Hence, when ATP regeneration is attenuated below the threshold which affects glucose uptake, we immediately observe a decrease in succinate productivity, thus explaining the triangular shaped succinate production profile under pyruvate flux-sum attenuation.

### Succinate production under flux-sum intensification

Flux-sum intensification analysis allowed us to observe the increase in succinate production when the flux-sum of several metabolites involved in redox reactions and the glyoxylate pathway were intensified. As the succinate-forming *FRD* consumes menaquinol to produce menaquinone, flux-sum intensification of such metabolites is expected to directly promote succinate production. It should be noted that the *i*AF1260 model accounts for two species of menquinones, demethylmenaquinone-8 and menaquinone-8, which were thus detected as potential targets for flux-sum intensification.

In the case of glyoxylate flux-sum intensification, its perturbation effect was understood by examining the TCA cycle with glyoxylate bypass. Under anaerobic condition, the metabolic flux through the TCA cycle is low: energy (ATP) is mainly regenerated by substrate-level phosphorylation rather than oxidative phosphorylation. Thus, increasing glyoxylate flux-sum will force a redistribution of fluxes away from the ethanol production pathway towards the TCA cycle. Consequently, the role of *ACALD* and *ALCD* in the ethanol production pathway as the major NAD regenerator is replaced by the succinate-producing *FRD* in the TCA cycle, similar to the metabolic state observed under pyruvate flux-sum attenuation. This strategy has also been demonstrated in a previous experimental study whereby the deletion of *iclR*, a repressor of the glyoxylate bypass operon *aceBAK* [30], increased glyoxylate pathway utilization and enhanced succinate production yield of more than eight fold [31].

### Minimizing acetate secretion under flux-sum perturbation

From the viewpoint of strain improvement, acetate secretion is usually undesirable as it inhibits cell growth and biochemical production [32, 33]. Hence, metabolite flux-sum perturbations for reducing acetate secretion will be of interest in this section. For flux-sum attenuation, only metabolites directly involved in acetate formation pathway, such as acetyl-CoA and acetylphosphate, were identified as targets. Previous experimental studies have also validated that the knockout of acetate kinase (*ackA*) or *pta*, which is equivalent to attenuating acetylphosphate flux-sum, can effectively reduce acetate formation [34, 35].

On the other hand, for flux-sum intensification, we identified about 30 potential metabolite targets, some of which are shown in Table 1. These metabolites were found to be mostly present in parallel biosynthetic pathways that compete with the acetate pathway for the same precursors. Thus, intensifying the flux-sum of key metabolites in these pathways may deprive the acetate pathway of metabolic fluxes, leading to reduced acetate formation. Examples of these metabolites include lactate from mixed acid fermentation pathway, isocitrate from TCA cycle, and ribulose 5-phosphate from the pentose phosphate pathway and 4-methyl-2-oxopentanoate from the leucine biosynthetic pathway. Some of these perturbation strategies to reduce acetate production have also been verified experimentally, e.g. isocitrate flux-sum intensification through deregulation of isocitrate lyase [36] and lactate flux-sum intensification by overexpression of lactate dehydrogenase [34].

An interesting and novel flux-sum intensification target for reducing acetate formation is 3-phosphoglycerate (3PG). As 3PG is an upstream precursor required for producing acetate, it is unexpected that increasing its turnover rate will attenuate acetate production. However, further examination of the flux distribution indicated that as the 3PG flux-sum increases, the pathway for glyoxylate catabolic process becomes activated. In short, glyoxylate catabolic process begins the synthesis of glyoxylate from isocitrate lyase and continues through a series of reactions, glyoxylate carboligase, tartronate semialdehyde reductase and glycerate kinase, where glyoxylate is converted back to 3PG. Since 3PG is a precursor for serine biosynthesis, the utilization of the glyoxylate metabolic pathway redistributes the fluxes such that cell growth is lowered. Consequently, the decrease of both energy requirement and resources for acetate synthesis leads to less flux through acetate kinase. Interestingly, a concomitant production of succinate was also observed as a result of glyoxylate bypass utilization. Thus, the intensification of 3PG flux-sum via overexpression of glyoxylate catabolic process can be a desirable metabolic engineering strategy for reducing acetate formation and increasing succinate production.

## Discussion

Recently, the application of flux-sum to a strain improvement study for vanillin production in *S. cerevisiae* was reported [37]. In that study, FSA was used to compute the minimum metabolite turnover which reflects the lower bound for the degree of resource allocation required to achieve a particular metabolic state. Subsequently, FSA was demonstrated to be a useful evaluation tool that provides insight into the cellular metabolism of engineered microbial strains from the metabolite perspective. Since the flux-sum concept can characterize resource distribution to various metabolites within the network, the application of FSA can be systematically utilized to identify metabolic engineering targets, rather than being used only as a supplementary tool to understand cellular metabolism. Accordingly, herein, we have proposed a systematic workflow to identify metabolic engineering targets using the FSA approach and demonstrated its applicability by applying it to *E. coli* for enhancing ethanol and succinate production, and reducing acetate formation. In general, this procedure can be simply applied to any other organism of interest whose genome-scale metabolic model is readily available. For example, we also applied the proposed FSA to *S. cerevisiae* using the *i*MM904 model [38] for succinate overproduction case and identified glyoxylate and acetyl-CoA as intensification and ethanol as attenuation targets, respectively (see Additional file 2 for details). Notably, the glyoxylate intensification strategy is very similar to the one identified in *E. coli* where the redirection of carbon flux towards glyoxylate cycle instead of the TCA cycle is beneficial for succinate overproduction. Collectively, such results reveal that the utilization of glyoxylate cycle could be a global strategy for succinate overproduction across different organisms.

The metabolic engineering targets identified using the metabolite-centric technique can be equivalent to the reaction targets obtained by the gene deletion-based approach. Thus, we compared the results of current work with that of OptKnock, OptReg and OptForce algorithms for the succinate overproduction case [19] to examine the uniqueness of the metabolite targets. Such comparison between reaction and metabolite targets revealed that although there are some comparable ones, not all the metabolites have an equivalent reaction (s) candidate (Table 2). For example, OptKnock consistently identified the deletion of *PTAr* and *ACKr*, corresponding to the attenuation of acetylphosphate and acetate, respectively. Similarly, the deletion/downregulation of *PFL*, as identified by OptKnock, OptReg and OptForce also correspond very well with the formate attenuation. Moreover, the overexpression of citrate synthase and aconitase in conjunction with the isocitrate dehydrogenase deletion may be an equivalent strategy to augment the glyoxylate pool as identified by FSA. However, despite such comparable results, the pyruvate flux-sum attenuation for succinate overproduction does not have many equivalent targets in reaction-centric algorithms, except *PYK* deletion, which is presumably due to pyruvate’s association with multiple reactions. Intensification of menaquinol/menaquinone is unique target identified by FSA which does not have equivalent reaction-centric targets. Such results clearly demonstrate the efficacy of FSA in identifying novel metabolite targets that could only have been found via the multiple reaction deletion analysis. In this sense, FSA can be considered as a useful tool to circumvent the much higher computational cost of perturbing the large number of reaction flux combinations. Nonetheless, the flux-sum analysis method is not completely superior to conventional gene/reaction deletion analysis as we have also identified unique reaction targets using the latter method. Such examples include the downregulation of malate dehydrogenase (MDH) identified by OptForce, which prevents the malate from getting converted into oxaloacetate and redirects the flux towards fumarate, and then to succinate. Hence, we propose FSA as a complementary procedure within the general constraints-based flux analysis framework for strain improvement studies.

**Table 2:** Comparison of metabolic engineering targets identified by various reaction-centric approaches and its equivalents in FSA for succinate overproduction in *E. coli*

Reaction-centric algorithm			FSA
Algorithm	# interventions	Targets	Equivalent target
OptKnock	Two	PFL (X), LDH (X)	PFL (X) corresponds to formate (↓)
	Three	ALCD (X), PFL (X), LDH (X)	PFL (X) corresponds to formate (↓)
		ALCD (X), PTA (X), ACK (X)	PTA (X) and ACK (X) corresponds to acetate (↓)
	Four	ALCD (X), PTA (X), ACK (X), PYK (X)	PTA (X) and ACK (X) correspond to acetate (↓). PYK (X) corresponds to pyruvate (↓)
		ALCD (X), PTA (X), ACK (X), TKT (X)	PTA (X) and ACK (X) correspond to acetate (↓)
OptReg	Two	PFL (X), PPC (↑)	PFL (X) corresponds to formate (↓)
	Three	PFL (X), PPC (↑), ALCD (↓)	PFL (X) corresponds to formate (↓)
	Four	PPC (↑), CS (↑), PDH (↓), ALCD (↓)	CS (↑) possibly correspond to isocitrate (↑)
OptForce	Two	PPC (↑), CS (↑)	CS (↑) possibly correspond to isocitrate (↑)
OptForce	Three	PPC (↑), CS (↑), MDH (↓)	CS (↑) and MDH (↓) possibly correspond to glyoxylate (↑) and isocitrate (↑)
		PPC (↑), ACONT (↑), MDH (↓)	ACONT (↑) and MDH (↓) possibly correspond to glyoxylate (↑) and isocitrate (↑)
OptForce	Four	PPC (↑), CS (↑), MDH (↓), PFL (↓)	CS (↑) and MDH (↓) possibly correspond to glyoxylate (↑) and isocitrate (↑). PFL (↓) corresponds to formate (↓)
		PPC (↑), ACONT (↑), MDH (↓), PFL (↓)	ACONT (↑) and MDH (↓) possibly correspond to glyoxylate (↑) and isocitrate (↑). PFL (↓) corresponds to formate (↓)

Results of OptKnock, OptReg and OptForce are reproduced from Ranganathan et al. [19]. X - Deletion, ↑ - Upregulation or Intensification and ↓ - Downregulation or Attenuation

While using FSA for metabolic engineering, following the identification of metabolite targets, we envisage two different approaches to manipulate the flux-sum of target metabolites. The first approach could be based on the genetic engineering of genes around the target metabolite in the metabolic network. For example, in order to intensify the 6-phospho-D-gluconate production levels, we can either overexpress the corresponding biosynthetic gene, glucose-6-phosphate dehydrogenase *(zwf*) or forcefully reroute excess carbon flux into the desired pentose phosphate pathway by deleting the phosphofructokinase (*pfk*) or phosphoglucose isomerase (*pgi*) genes from the parallel glycolytic pathway. In the second approach, more intuitive strategies such as the use of antimetabolites or co-feeding of certain pathway intermediates in the culture media could possibly restrict or enhance the target metabolites’ turnover rates. Although the use of such compounds to manipulate the flux-sum of a particular metabolite has never been attempted before, it could still be an interesting option to explore.

## Conclusion

In this study, we successfully identified potential metabolite targets that can enhance the cellular physiology of *E. coli* using the FSA method developed in our previous work [22]. The original FSA framework was modified to elucidate changes in cellular metabolism under flux-sum perturbation, leading to the identification of metabolic engineering targets for strain improvement. The *in silico* simulation presented results that were highly consistent with previous wet-lab experimental observations, and also novel findings that could be validated in future works. In addition, comparison with reaction targets identified by conventional gene/reaction deletion analysis shows that FSA has the capability to identify unique metabolite targets. Thus, the application of FSA complemented with conventional gene deletion analysis will provide researchers with a wider choice of potential metabolic engineering targets for stain improvement, which will be beneficial to the field of systems metabolic engineering.

## Methods

### Constraints-based flux analysis

Constraints-based flux analysis is an *in silico* method that simulates cellular metabolism such that a hypothetical cellular objective, usually cell growth, is maximized under stoichiometric and reaction capacity constraints [39–41]. The mathematical formulation is in the form of a linear programming (LP) problem as follows:

1 $$ \max\ {Z}_1={\displaystyle \sum_j{c}_j{v}_j} $$

Subject to:

$$ {\displaystyle \sum_j{S}_{ij}{v}_j=0} $$

$$ {a}_j\le {v}_j\le {\beta}_j $$

where *c*_*j*_ is the coefficient of the desired cellular objective to be maximized, *v*_*j*_ is the flux of reaction *j* and *S*_*ij*_ indicates the stoichiometric coefficient of metabolite *i* involved in reaction *j*. Values of *α*_*j*_ and *β*_*j*_ can be specified based on experimental measurements or any hypothetical flux perturbation. Solving Eq. (1) by setting *Z*_1_ to be equal to cell growth rate, without any flux perturbation constraint, yields the wild-type (WT) metabolic flux distribution.

### Quantification of metabolite flux-sum

Conventional constraints-based flux analysis does not provide a means to quantify the degree of individual metabolite utilization. Hence, we introduced the flux-sum concept to quantify metabolite turnover rates [22, 23]. Under the steady-state flux balanced condition, the consumption and generation rates for any metabolite are equal.

i.e $$ {\displaystyle \sum_j{\left|{S}_{ij}{v}_j\right|}_{consumption}}={\displaystyle \sum_j{\left|{S}_{ij}{v}_j\right|}_{generation}} $$

Thus, the flux-sum of any intermediate metabolite *i*, Φ_*i*_, can be calculated as half the sum of the absolute rate of all reactions consuming or producing the metabolite:

2 $$ {\Phi}_i=0.5{\displaystyle \sum_j\left|{S}_{ij}{v}_j\right|} $$

Unlike the mathematical formulation of the basic constraints-based flux analysis, the flux-sum expression is nonlinear due to the modulus operator and directly imposing constraints on the flux-sum expression will result in a nonlinear problem. Hence, additional constraints are introduced to recast the problem to a linear integer form as discussed previously [22]:

$$ {\Phi}_i=0.5{\displaystyle \sum_j\left({g}_{ij}^{+}+{g}_{ij}^{-}\right)} $$

$$ \mathrm{where}\kern1em {S}_{ij}{v}_j={g}_{ij}^{+}-{g}_{ij}^{-} $$

$$ {g}_{ij}^{+}\ge 0\kern1em ;\kern1em {g}_{ij}^{-}\ge 0 $$

$$ {g}_{ij}^{+}\le {I}_{ij}^{+}\cdot M\kern1em ;\kern1em {g}_{ij}^{-}\le {I}_{ij}^{-}\cdot M $$

$$ {I}_{ij}^{+}\in \left\{0,1\right\}\kern1em ;\kern1em {I}_{ij}^{-}\in \left\{0,1\right\} $$

$$ {I}_{ij}^{+}+{I}_{ij}^{-}=1 $$

The positive variables, *g*_*ij*_^+^ and *g*_*ij*_^−^, refer to the generation and consumption components of metabolite *i* due to reaction *j*, respectively. Binary variables, *I*_*ij*_^+^ and *I*_*ij*_^−^, serve as switches to turn the generation and consumption components on and off such that only one of the component is active, effected by the *I*_*ij*_^+^ + *I*_*ij*_^−^ = 1 constraint. Big *M* can be an arbitrarily large flux value, e.g. 1000 mmol/gDCW-hr.

### Flux-sum analysis for identifying metabolic engineering targets

To identify the attenuation and intensification metabolite targets which lead to the enhanced production of desired compound, we first need to quantify the flux-sum of all metabolites in the wild-type strain. This can be determined by first solving the constraints-based flux analysis problem (Eq. 1) with biomass maximization as objective, and then substituting the resulting flux distribution into Eq. (2). Next, we need to solve the below mentioned MILP problem to identify the flux-sum maxima and minima to determine the feasible ranges of individual metabolite flux-sum such that they can be attenuated or intensified within this limit:

3 $$ \max / \min \kern1em {\Phi}_i=0.5{\displaystyle \sum_j\left({g}_{ij}^{+}+{g}_{ij}^{-}\right)}\ \mathrm{f}\mathrm{o}\mathrm{r}\ \mathrm{f}\mathrm{iven}\ \mathrm{metabolite}i $$

$$ \mathrm{Subject}\ \mathrm{t}\mathrm{o}: $$

$$ {\displaystyle \sum_j{S}_{ij}{v}_j}=0 $$

$$ {\alpha}_j\le {v}_j\le {\beta}_j $$

$$ {S}_{ij}{v}_j={g}_{ij}^{+}-{g}_{ij}^{-} $$

$$ {g}_{ij}^{+}\ge 0\kern1em ;\kern1em {g}_{ij}^{-}\ge 0 $$

$$ {g}_{ij}^{+}\le {I}_{ij}^{+}\cdot M\kern1em ;\kern1em {g}_{ij}^{-}\le {I}_{ij}^{-}\cdot M $$

$$ {I}_{ij}^{+}\in \left\{0,1\right\}\kern1em ;\kern1em {I}_{ij}^{-}\in \left\{0,1\right\} $$

$$ {I}_{ij}^{+}+{I}_{ij}^{-}=1 $$

Once the reference flux-sum values are established, i.e. wild-type, maxima and minima values, we then solve the below mentioned MILP problem to analyze the effects of perturbing a particular metabolite’s turnover rate on cellular growth.

4 $$ \max \kern1em {v}_{biomass} $$

Subject to:

$$ {\displaystyle \sum_j{S}_{ij}{v}_j}=0 $$

$$ {\alpha}_j\le {v}_j\le {\beta}_j $$

$$ {S}_{ij}{v}_j={g}_{ij}^{+}-{g}_{ij}^{-} $$

$$ {g}_{ij}^{+}\ge 0\kern1em ;\kern1em {g}_{ij}^{-}\ge 0 $$

$$ {g}_{ij}^{+}\le {I}_{ij}^{+}\cdot M\kern1em ;\kern1em {g}_{ij}^{-}\le {I}_{ij}^{-}\cdot M $$

$$ {I}_{ij}^{+}\in \left\{0,1\right\}\kern1em ;\kern1em {I}_{ij}^{-}\in \left\{0,1\right\} $$

$$ {I}_{ij}^{+}+{I}_{ij}^{-}=1 $$

$$ \left(\mathbf{C}\mathbf{1}\right):\kern0.5em 0.5{\displaystyle \sum_j{g}_{ij}^{+}+{g}_{ij}^{-}}\le {\Phi}_i^{\min }+{k}_{att}\left({\Phi}_i^{WT}-{\Phi}_i^{\min}\right) $$

**OR**

$$ \left(\mathbf{C}\mathbf{2}\right):\kern0.5em 0.5{\displaystyle \sum_j{g}_{ij}^{+}+{g}_{ij}^{-}}\ge {\Phi}_i^{WT}+{k}_{\mathrm{int}}\left({\Phi}_i^{\max }-{\Phi}_i^{WT}\right) $$

where constraint (C1) and (C2) is applicable for attenuation and intensification problems, respectively. Parameters *k*_*att*_ and *k*_*int*_ are gradually varied between 0 and 1 in steps of 0.1 to analyze the effect of metabolite attenuation between minimal and wild-type values, and intensification of metabolite turnover between the wild-type and maximal values, respectively.

Finally, the objective value obtained from the solution of Eq. (4) is used as the lower limit for cell growth in the fourth step whereby (Eq. 4) is solved again with the targeted worst-case scenario as the objective function. For example, if we aim to increase succinate production rate, the respective flux can be minimized to evaluate the worst case scenario. The corresponding mathematical formulation is as follows:

$$ \min \kern1em {v}_{EX\_ succ} $$

Subject to:

$$ {v}_{biomass}\ge {B}_{i,k} $$

$$ {\displaystyle \sum_j{S}_{ij}{v}_j}=0 $$

$$ {\alpha}_j\le {v}_j\le {\beta}_j $$

$$ {S}_{ij}{v}_j={g}_{ij}^{+}-{g}_{ij}^{-} $$

$$ {g}_{ij}^{+}\ge 0\kern1em ;\kern1em {g}_{ij}^{-}\ge 0 $$

$$ {g}_{ij}^{+}\le {I}_{ij}^{+}\cdot M\kern1em ;\kern1em {g}_{ij}^{-}\le {I}_{ij}^{-}\cdot M $$

$$ {I}_{ij}^{+}\in \left\{0,1\right\}\kern1em ;\kern1em {I}_{ij}^{-}\in \left\{0,1\right\} $$

$$ {I}_{ij}^{+}+{I}_{ij}^{-}=1 $$

$$ \left(\mathbf{C}\mathbf{1}\right):\kern0.5em 0.5{\displaystyle \sum_j{g}_{ij}^{+}+{g}_{ij}^{-}}\le {\Phi}_i^{\min }+{k}_{\mathrm{att}}\left({\Phi}_i^{WT}-{\Phi}_i^{\min}\right) $$

**OR**

$$ \left(\mathbf{C}\mathbf{2}\right):\kern0.5em 0.5{\displaystyle \sum_j{g}_{ij}^{+}+{g}_{ij}^{-}}\ge {\Phi}_i^{WT}+{k}_{\mathrm{int}}\left({\Phi}_i^{\max }-{\Phi}_i^{WT}\right) $$

where *B*_*ik*_ is the maximum biomass obtainable while solving problem (Eq. 4) for *i*^th^ metabolite at *k*^th^ attenuation/intensification levels. Here, it should be noted that we problem (Eq. 4) in two steps whereby first maximizing biomass objective and then maximizing/minimizing targeted worst-case scenario with minimum biomass constrained at the value obtained in previous step to make sure that there is no other alternative optima present.

### Implementation of flux-sum analysis

The FSA procedure is applied to the *i*AF1260 metabolic model of *E. coli* [42] to investigate the effects of flux-sum perturbation on ethanol, succinate and acetate production. All *in silico* simulations were carried out based on a glucose uptake rate of 1 g/gDCW-hr, such that all flux values can also be interpreted as yield in mmol/g glucose or g/g glucose. Since by-product formation in *E. coli* typically occurs under the anaerobic condition, we also constrained oxygen uptake rate to zero. All the optimization problems were solved using the GAMS IDE software version 22.4 [43] with IBM ILOG CPLEX solver.

## Additional files

Additional file 1:
**Ethanol, acetate and succinate production profiles in** ***Escherichia coli*** **under metabolite flux-sum attenuation/intensification.** (DOC 129 kb). Additional file 2:
**Attenuation/intensification targets for succinate overproduction in Saccharomyces cerevisiae.** (DOC 62 kb).

## References

[1] Lee SY, Lee DY, Kim TY (2005). Systems biotechnology for strain improvement. Trends Biotechnol.

[2] Otero JM, Nielsen J (2010). Industrial systems biology. Biotechnol Bioeng.

[3] Park JH, Lee SY (2008). Towards systems metabolic engineering of microorganisms for amino acid production. Curr Opin Biotechnol.

[4] Kim TY, Sohn SB, Kim YB, Kim WJ, Lee SY (2012). Recent advances in reconstruction and applications of genome-scale metabolic models. Curr Opin Biotechnol.

[5] Monk J, Nogales J, Palsson BO (2014). Optimizing genome-scale network reconstructions. Nat Biotechnol.

[6] Oberhardt MA, Palsson BO, Papin JA (2009). Applications of genome-scale metabolic reconstructions. Mol Syst Biol.

[7] Lakshmanan M, Koh G, Chung BK, Lee DY (2014). Software applications for flux balance analysis. Brief Bioinform.

[8] Lewis NE, Nagarajan H, Palsson BO (2012). Constraining the metabolic genotype-phenotype relationship using a phylogeny of *in silico* methods. Nat Rev Microbiol.

[9] Bordbar A, Monk JM, King ZA, Palsson BO (2014). Constraint-based models predict metabolic and associated cellular functions. Nat Rev Genet.

[10] Alper H, Jin YS, Moxley JF, Stephanopoulos G (2005). Identifying gene targets for the metabolic engineering of lycopene biosynthesis in Escherichia coli. Metab Eng.

[11] Lee SJ, Lee DY, Kim TY, Kim BH, Lee J, Lee SY (2005). Metabolic engineering of Escherichia coli for enhanced production of succinic acid, based on genome comparison and in silico gene knockout simulation. Appl Environ Microbiol.

[12] Burgard AP, Pharkya P, Maranas CD (2003). Optknock: a bilevel programming framework for identifying gene knockout strategies for microbial strain optimization. Biotechnol Bioeng.

[13] Pharkya P, Maranas CD (2006). An optimization framework for identifying reaction activation/inhibition or elimination candidates for overproduction in microbial systems. Metab Eng.

[14] Pharkya P, Burgard AP, Maranas CD (2004). OptStrain: a computational framework for redesign of microbial production systems. Genome Res.

[15] Patil KR, Rocha I, Forster J, Nielsen J (2005). Evolutionary programming as a platform for *in silico* metabolic engineering. BMC Biochem..

[16] Lee KH, Park JH, Kim TY, Kim HU, Lee SY (2007). Systems metabolic engineering of *Escherichia coli* for L-threonine production. Mol Syst Biol.

[17] Tepper N, Shlomi T (2010). Predicting metabolic engineering knockout strategies for chemical production: accounting for competing pathways. Bioinformatics.

[18] Choi HS, Lee SY, Kim TY, Woo HM (2010). In silico identification of gene amplification targets for improvement of lycopene production. Appl Environ Microbiol.

[19] Ranganathan S, Suthers PF, Maranas CD (2010). OptForce: an optimization procedure for identifying all genetic manipulations leading to targeted overproductions. PLoS Comput Biol.

[20] Lakshmanan M, Chung BK, Liu C, Kim S-W, Lee D-Y (2013). Cofactor modification analysis: A computational framework to identify cofactor specificity engineering targets for strain improvement. J Bioinform Comput Biol.

[21] Long MR, Ong WK, Reed JL (2015). Computational methods in metabolic engineering for strain design. Curr Opin Biotechnol.

[22] Chung BK, Lee DY (2009). Flux-sum analysis: a metabolite-centric approach for understanding the metabolic network. BMC Syst Biol.

[23] Kim PJ, Lee DY, Kim TY, Lee KH, Jeong H, Lee SY (2007). Metabolite essentiality elucidates robustness of *Escherichia coli* metabolism. Proc Natl Acad Sci U S A.

[24] Lee KY, Park JM, Kim TY, Yun H, Lee SY (2010). The genome-scale metabolic network analysis of *Zymomonas mobilis* ZM4 explains physiological features and suggests ethanol and succinic acid production strategies. Microb Cell Fact.

[25] Trinh CT, Unrean P, Srienc F (2008). Minimal *Escherichia coli* cell for the most efficient production of ethanol from hexoses and pentoses. Appl Environ Microbiol.

[26] Shams Yazdani S, Gonzalez R (2008). Engineering *Escherichia coli* for the efficient conversion of glycerol to ethanol and co-products. Metab Eng.

[27] Underwood SA, Zhou S, Causey TB, Yomano LP, Shanmugam KT, Ingram LO (2002). Genetic changes to optimize carbon partitioning between ethanol and biosynthesis in ethanologenic *Escherichia coli*. Appl Environ Microbiol.

[28] Fuhrer T, Sauer U (2009). Different biochemical mechanisms ensure network-wide balancing of reducing equivalents in microbial metabolism. J Bacteriol.

[29] Ohta K, Beall DS, Mejia JP, Shanmugam KT, Ingram LO (1991). Genetic improvement of *Escherichia coli* for ethanol production: chromosomal integration of *Zymomonas mobilis* genes encoding pyruvate decarboxylase and alcohol dehydrogenase II. Appl Environ Microbiol.

[30] Gui L, Sunnarborg A, Pan B, LaPorte DC (1996). Autoregulation of *iclR*, the gene encoding the repressor of the glyoxylate bypass operon. J Bacteriol.

[31] Wang Q, Chen X, Yang Y, Zhao X (2006). Genome-scale *in silico* aided metabolic analysis and flux comparisons of *Escherichia coli* to improve succinate production. Appl Microbiol Biotechnol.

[32] Eiteman MA, Altman E (2006). Overcoming acetate in *Escherichia coli* recombinant protein fermentations. Trends Biotechnol.

[33] De Mey M, De Maeseneire S, Soetaert W, Vandamme E (2007). Minimizing acetate formation in *E. coli* fermentations. J Ind Microbiol Biotechnol.

[34] Yang YT, Aristidou AA, San KY, Bennett GN (1999). Metabolic flux analysis of *Escherichia coli* deficient in the acetate production pathway and expressing the *Bacillus subtilis* acetolactate synthase. Metab Eng.

[35] Diaz-Ricci JC, Regan L, Bailey JE (1991). Effect of alteration of the acetic acid synthesis pathway on the fermentation pattern of *Escherichia coli*. Biotechnol Bioeng.

[36] Farmer WR, Liao JC (1997). Reduction of aerobic acetate production by *Escherichia coli*. Appl Environ Microbiol.

[37] Brochado AR, Matos C, Moller BL, Hansen J, Mortensen UH, Patil KR (2010). Improved vanillin production in baker’s yeast through *in silico* design. Microb Cell Fact.

[38] Mo ML, Palsson BO, Herrgard MJ (2009). Connecting extracellular metabolomic measurements to intracellular flux states in yeast. BMC Syst Biol.

[39] Llaneras F, Pico J (2008). Stoichiometric modelling of cell metabolism. J Biosci Bioeng.

[40] Raman K, Chandra N (2009). Flux balance analysis of biological systems: applications and challenges. Brief Bioinform.

[41] Oberhardt MA, Chavali AK, Papin JA (2009). Flux balance analysis: interrogating genome-scale metabolic networks. Methods Mol Biol.

[42] Feist AM, Henry CS, Reed JL, Krummenacker M, Joyce AR, Karp PD (2007). A genome-scale metabolic reconstruction for *Escherichia coli* K-12 MG1655 that accounts for 1260 ORFs and thermodynamic information. Mol Syst Biol.

[43] Brooke A, Kendrick D, Meeraus A, Raman R (1998) GAMS - A user’s guide. In. GAMS Development Corporation, Washington, D.C

