# Supplementary material for: Flux-sum analysis identifies metabolite targets for strain improvement
Source: BMC Syst Biol. 2015 Oct 29;9:73. doi: 10.1186/s12918-015-0198-3 (PMC4625974; doi:10.1186/s12918-015-0198-3)
Supplement: Additional file 1: — Ethanol, acetate and succinate production profiles in Escherichia coli under metabolite flux-sum attenuation/intensification. (DOC 129 kb). [file 12918_2015_198_MOESM1_ESM.doc]

**Additional file 1**

**Flux-sum analysis identifies metabolite targets for strain improvement**

## Meiyappan Lakshmanan, Tae Yong Kim, Bevan KS Chung , Sang Yup Lee, Dong-Yup Lee


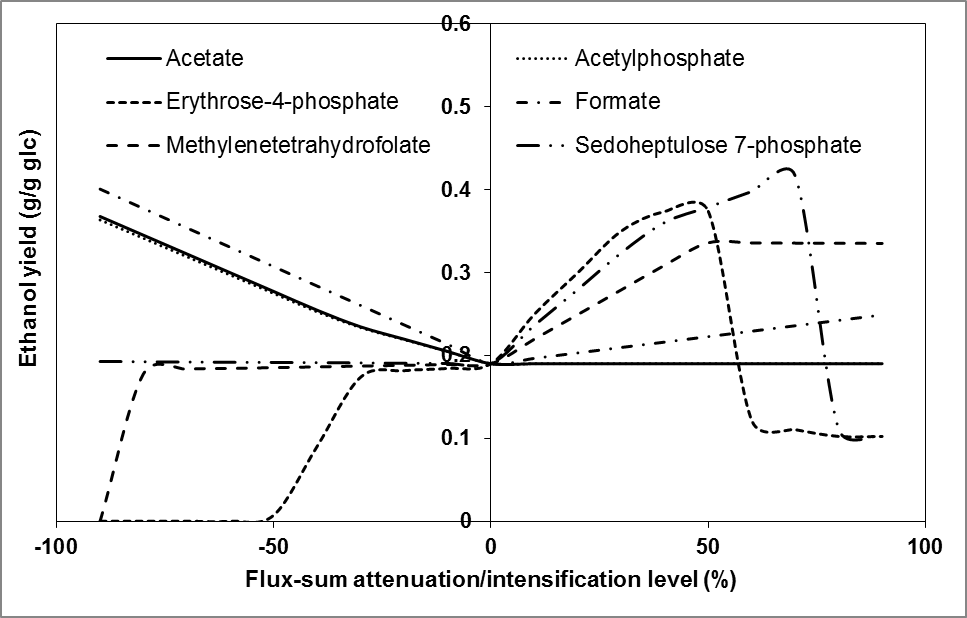


## Figure A1 – Ethanol production profile under metabolite flux-sum attenuation/intensification

The left quadrant of the figure shows the attenuation region whereas the right side corresponds to the intensification.


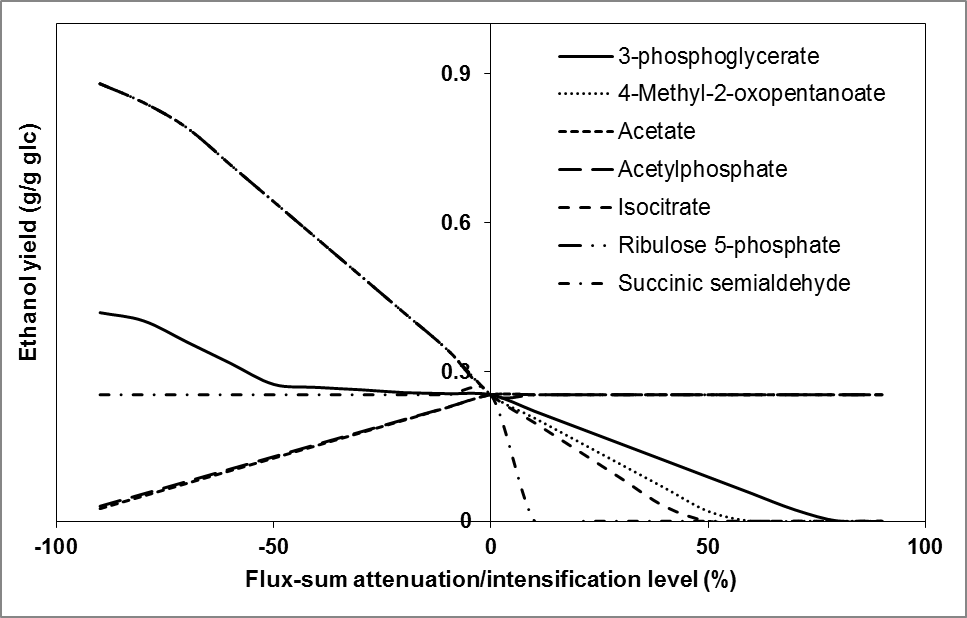


## Figure A2 – Acetate production profile under metabolite flux-sum attenuation/intensification

The left quadrant of the figure shows the attenuation region whereas the right side corresponds to the intensification.


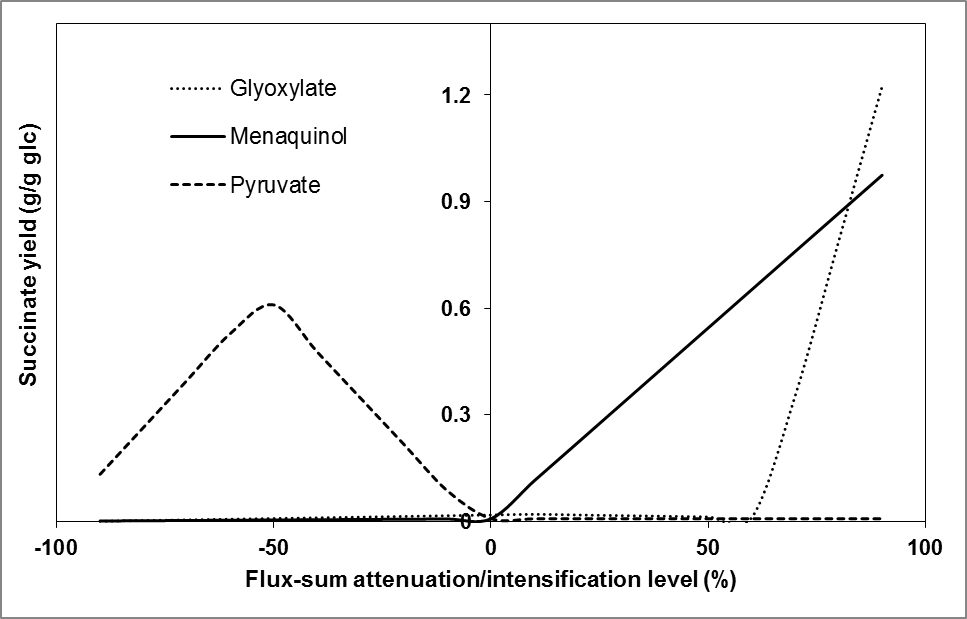


## Figure A3 – Succinate production profile under metabolite flux-sum attenuation/intensification

The left quadrant of the figure shows the attenuation region whereas the right side corresponds to the intensification.
