# Supplementary material for: Flux-sum analysis identifies metabolite targets for strain improvement
Source: BMC Syst Biol. 2015 Oct 29;9:73. doi: 10.1186/s12918-015-0198-3 (PMC4625974; doi:10.1186/s12918-015-0198-3)
Supplement: Additional file 2: — Attenuation/intensification targets for succinate overproduction in Saccharomyces cerevisiae. (DOC 62 kb). [file 12918_2015_198_MOESM2_ESM.doc]

**Additional file 2**

**Flux-sum analysis identifies metabolite targets for strain improvement**

## Meiyappan Lakshmanan, Tae Yong Kim, Bevan KS Chung , Sang Yup Lee, Dong-Yup Lee

## Application of FSA to *S. cerevisiae*

It is well known that the cellular metabolism of different microbes vary significantly due to several factors such as network organization, nutrient uptake ability and cofactor regeneration. As such, the turnover of various metabolites, even the ones from central metabolism, differs remarkably across microbes . Therefore, in order to examine whether the metabolic network organization significantly influence the flux-sum targets, we performed FSA in *Saccharomyces cerevisiae* using the *i*MM904 model for succinate overproduction and compared the results with that of *E. coli* (Table A1). FSA identified ethanol as the solitary attenuation target for obvious reasons, and glyxolyate and acetyl-CoA as amplification targets (Figure A4). Notably, the glyoxylate intensification strategy is very similar to the one demonstrated in the previous experimental study where the deletion of TCA cycle genes redirected the carbon flux towards glyoxylate cycle, and thus leading to significant overproduction of succinate . Collectively, such comparative analysis reveals that the flux-sum perturbation targets are not always same across different kingdoms as the turnover rates of various metabolites is unique to each organism.

## References

1. Lee KY, Park JM, Kim TY, Yun H, Lee SY: **The genome-scale metabolic network analysis of *Zymomonas mobilis* ZM4 explains physiological features and suggests ethanol and succinic acid production strategies**. *Microb Cell Fact* 2010, **9**:94.

2. Mo ML, Palsson BO, Herrgard MJ: **Connecting extracellular metabolomic measurements to intracellular flux states in yeast**. *BMC Syst Biol* 2009, **3**:37.

3. Raab AM, Gebhardt G, Bolotina N, Weuster-Botz D, Lang C: **Metabolic engineering of Saccharomyces cerevisiae for the biotechnological production of succinic acid**. *Metabolic Engineering* 2010, **12**(6):518-525.

4. Verwaal R, Wu L, Damveld RA, Sagt CMJ: **Succinate production in a eukaryotic cell**. In*.*, vol. WO20120165569 A1. US; 2012.

## Table A1 - List of metabolic engineering targets in *S. cerevisiae*

| **Target metabolites** | **Flux-sum perturbation** | **Experimental validation** |
| --- | --- | --- |
|  |  |  |
| Ethanol | Attenuation |  |
| Glyoxylate | Intensification |  |
| Acetyl-CoA | Intensification | N.A. |


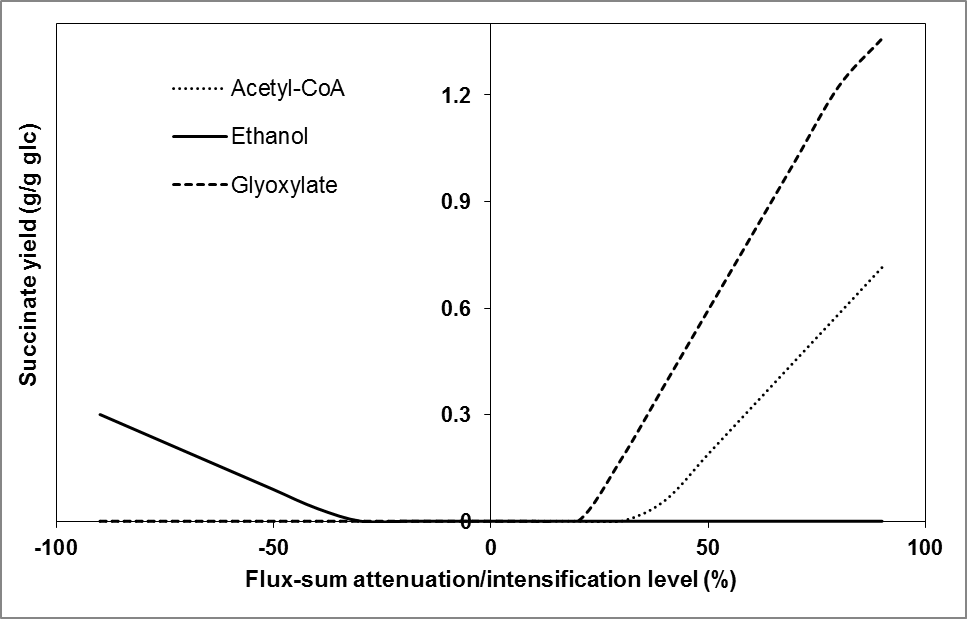


## Figure A4 – Succinate production profile under metabolite flux-sum attenuation/intensification

The left quadrant of the figure shows the attenuation region whereas the right side corresponds to the intensification.
